# Phenotypic and Genomic Variability of Serial Peri-Lung Transplantation *Pseudomonas aeruginosa* Isolates From Cystic Fibrosis Patients

**DOI:** 10.3389/fmicb.2021.604555

**Published:** 2021-04-07

**Authors:** Rucha Datar, Andreu Coello Pelegrin, Sylvain Orenga, Valérie Chalansonnet, Caroline Mirande, Jill Dombrecht, John D. Perry, Audrey Perry, Herman Goossens, Alex van Belkum

**Affiliations:** ^1^BioMérieux, La Balme les Grottes, France; ^2^Laboratory of Medical Microbiology, Faculty of Medicine and Health Sciences, Vaccine and Infectious Disease Institute, University of Antwerp, Antwerp, Belgium; ^3^Applied Maths, BioMérieux, Sint-Martens-Latem, Belgium; ^4^Freeman Hospital, Newcastle upon Tyne, United Kingdom

**Keywords:** *Pseudomonas aeruginosa*, lung transplantation, antimicrobial resistance, cystic fibrosis, whole genome sequencing

## Abstract

Cystic fibrosis (CF) represents one of the major genetic and chronic lung diseases affecting Caucasians of European descent. Patients with CF suffer from recurring infections that lead to further damage of the lungs. Pulmonary infection due to *Pseudomonas aeruginosa* is most prevalent, further increasing CF-related mortality. The present study describes the phenotypic and genotypic variations among 36 *P. aeruginosa* isolates obtained serially from a non-CF and five CF patients before, during and after lung transplantation (LTx). The classical and genomic investigation of these isolates revealed a common mucoid phenotype and only subtle differences in the genomes. Isolates originating from an individual patient shared ≥98.7% average nucleotide identity (ANI). However, when considering isolates from different patients, substantial variations in terms of sequence type (ST), virulence factors and antimicrobial resistance (AMR) genes were observed. Whole genome multi-locus sequence typing (MLST) confirmed the presence of unique STs per patient regardless of the time from LTx. It was supported by the monophyletic clustering found in the genome-wide phylogeny. The antibiogram shows that ≥91.6% of the isolates were susceptible to amikacin, colistin and tobramycin. For other antibiotics from the panel, isolates frequently showed resistance. Alternatively, a comparative analysis of the 36 *P. aeruginosa* isolates with 672 strains isolated from diverse ecologies demonstrated clustering of the CF isolates according to the LTx patients from whom they were isolated. We observed that despite LTx and associated measures, all patients remained persistently colonized with similar isolates. The present study shows how whole genome sequencing (WGS) along with phenotypic analysis can help us understand the evolution of *P. aeruginosa* over time especially its antibiotic resistance.

## Introduction

Cystic fibrosis (CF) is one of the most common and ultimately lethal autosomal recessive diseases, which primarily affects Caucasians of European descent (Cutting, 2015). Lung diseases are the main cause of morbidity and mortality among CF patients, especially those leading to respiratory failure caused by extensive lung damage, such as bronchiectasis or chronic endobronchial infections (Sanders and Fink, 2016). For patients with end-stage CF, lung transplantation (LTx) is the only viable option, as there is a 50% survival rate 10-year post-LTx (Snell et al., 2017).

The respiratory tract microbiota of CF is diverse and the most prevalent pathogens include *Pseudomonas aeruginosa*, *Staphylococcus aureus*, *Haemophilus influenzae*, *Burkholderia cepacia* complex (BCC), *Stenotrophomonas maltophilia*, *Achromobacter xylosoxidans*, and non-tuberculous mycobacteria (NTM). The exact incidence for each bacterial species has changed over the past years and continues to change, sometimes according to the geographic locale of the patients (Salsgiver et al., 2016). Frequent antibiotic therapies for patients with CF tend to select for antibiotic resistant variants. Multidrug-resistant (MDR) *P. aeruginosa* is one of the major end-stage bacterial species among CF patients, as even after antibiotic treatment it frequently recurs and establishes chronic infection. Such strains also display evolution of phenotypic and genomic characteristics over time (Winstanley et al., 2016; Stefani et al., 2017).

As *P. aeruginosa* is highly versatile in its pathogenicity and has significant genomic plasticity, assessing the whole genome sequence and identifying antimicrobial resistance (AMR) genes could be very useful to gain insights into the emergence of resistance against currently used antimicrobials. Population-level analysis and comparative genomics can help us to identify the co-existence of diverse strains during chronic illnesses and how they differ genotypically (Chung et al., 2012; Celli et al., 2015). High resolution genomics can enable us to better understand the high-risk clones (HRC) and segregate them as MDR or extensively-drug resistant or pan-drug resistant (Magiorakos et al., 2012). Amongst the many *P. aeruginosa* HRC studied, sequence type (ST) 235 is the most widespread lineage across the world.

The aim of this study is to evaluate how whole genome sequencing (WGS) and bio-informatic data analysis could assist in evaluating the impact of LTx on the diversity and antibiotic resistance profile of resident *P. aeruginosa* isolates. We studied culture based phenotypes of the isolates in combination with WGS to analyze the local epidemiology, evolution and resistome of *P. aeruginosa* isolates. They were serially obtained from five CF patients and a non-CF bronchiectasis patient, who all underwent LTx. Genomic data were analyzed versus a large control panel of *P. aeruginosa* strains (*n* = 672) from different ecological and clinical niches.

## Materials and Methods

### Sample Collection and Isolation of *Pseudomonas aeruginosa*

For the investigation of isolates from the same LTx patients, obtained at different time points, before, during and after LTx, serial peri-lung transplantation (SPLT) *P. aeruginosa* isolates, data from the six patients who underwent LTx during the years 2006–2017 were retrieved from the Freeman Hospital in Newcastle upon Tyne (United Kingdom). These patients were in the age group of 24–42 years at the time of LTx. For each patient, sampling time range from 6 to 22 months. At various intervals (i.e., before, during and after LTx) either broncho-alveolar lavage (BAL) or sputum specimens were collected for microbiological assessment at the Freeman Hospital. We selected all colony variants (mucoid, non-mucoid, large, small, etc.) isolated at a particular time interval. If only one isolate was selected for testing, then it means that all colonies of *P. aeruginosa* showed a consistent appearance. We cannot of course discount the possibility that colonies that look identical may have different phenotypes in terms of, for example, antimicrobial susceptibility. In total, 36 *P. aeruginosa* isolates belonging to five CF patients and a non-CF bronchiectasis patient, were isolated and preserved at −80°C until further analysis (Table 1). A sub-selection of 12 isolates, one pre- and one post-LTx from each of the six patients was used for full antibiotic susceptibility characterization (star marked in Table 1). Three isolates were used as quality controls throughout the study: one CLSI/EUCAST quality control strain (ATCC: 27853) and 2 isolates resistant to carbapenems by impermeability (BioMérieux collection numbers: API: 1508289 and API: 9208013).

**TABLE 1:** The 36 *Pseudomonas aeruginosa* isolates represented on the basis of the patient number.

Isolate ID	Specimen type	Collection date^a^	Patient ID	Time to LTx (months)^b^
5627*	BAL	6/2/2015	Patient 1	4 Pre-LTx
5852	BAL	10/7/2015	Patient 1	0
6182	BAL	4/18/2016	Patient 1	6 Post-LTx
6984*	BAL	8/1/2017	Patient 1	10 Post-LTx
5892*	BAL	11/3/2015	Patient 2	12 Pre-LTx
6291	BAL	7/7/2016	Patient 2	4 Pre-LTx.
6543	BAL	11/5/2016	Patient 2	0
6544	BAL	11/5/2016	Patient 2	0
6820*	BAL	5/17/2017	Patient 2	6 Post-LTx
2755*	BAL	7/14/2011	Patient 3	11 Pre-LTx
2756	BAL	7/14/2011	Patient 3	11 Pre-LTx
3472	BAL	6/29/2012	Patient 3	0
3473	BAL	6/29/2012	Patient 3	0
3856	Sputum	1/7/2013	Patient 3	7 Post-LTx
3857	Sputum	1/7/2013	Patient 3	7 Post-LTx
4007*	BAL	4/9/2013	Patient 3	10 Post-LTx
3731*	BAL	11/2/2012	Patient 4	3 Pre-LTx
3732	BAL	11/2/2012	Patient 4	3 Pre-LTx
3734	BAL	11/2/2012	Patient 4	3 Pre-LTx
3735	BAL	11/2/2012	Patient 4	3 Pre-LTx
3736	BAL	11/2/2012	Patient 4	3 Pre-LTx
3940	BAL	2/22/2013	Patient 4	0
3941	BAL	2/22/2013	Patient 4	0
3942	BAL	2/22/2013	Patient 4	0
4139*	BAL	6/3/2013	Patient 4	3,5 Post-LTx
4836*	BAL	4/25/2014	Patient 5	12 Pre-LTx
4837	BAL	4/25/2014	Patient 5	12 Pre-LTx
3312	BAL	9/3/2014	Patient 5	8 Pre-LTx
5579	BAL	5/14/2015	Patient 5	0
3927*	BAL	10/10/2015	Patient 5	5 Post-LTx
160*	BAL	7/26/2006	Patient 6	2 Pre-LTx
179	BAL	9/3/2006	Patient 6	0
810	BAL	4/11/2008	Patient 6	6 Post-LTx
811	BAL	4/11/2008	Patient 6	6 Post-LTx
1118*	BAL	11/7/2008	Patient 6	12 Post-LTx
1119	BAL	11/7/2008	Patient 6	12 Post-LTx

Two strains (star marked) per patient were selected and a panel of 12 *P. aeruginosa* isolates was created as a representative subset of the 36 strains. BAL, Bronchoalveolar lavage; NA, Not available; LTx, Lung transplantation. *Samples selected as a representative subset of the *P. aeruginosa* panel. ^*a*^Month/day/year format. ^*b*^Zero (0) represent samples obtained during the lung transplantation operation.

### Phenotypic Characterization of Serial Peri-Lung Transplantation *Pseudomonas aeruginosa* Isolates

#### Identification of SPLT *Pseudomonas aeruginosa* Isolates

The 36 *P. aeruginosa* isolates obtained were revived from glycerol stocks by streaking on Columbia agar + 5% sheep blood (COS agar) and incubated at 37°C for 24 h. The identity of the isolates was validated using mass spectrometry (Matrix Assisted Laser Desorption Ionization–Time of Flight MS) using VITEK^®^ MS (BioMérieux).

#### Antibiotic Susceptibility Testing Profiles of SPLT *Pseudomonas aeruginosa* Isolates

The 36 isolates were tested for their resistance/susceptibility profile against an array of antimicrobials by disk diffusion assay (Oxoid) according to CLSI (2017) guidelines. Additionally, minimum inhibitory concentrations (MIC) of a sub-selection of 12 isolates were defined by broth micro-dilution (BMD) assays to validate the results from disk diffusion assays. The antibiotic susceptibility testing (AST) profiling by BMD involved assessment using eight antibiotics viz. amikacin (AK), ciprofloxacin (CIP), levofloxacin (LEV), tobramycin (TOB), colistin (COL), imipenem (IMP), aztreonam (ATM), and ceftolozane-tazobactam (CZT). For BMD, the antibiotics were diluted using Mueller Hinton Broth (MHB) forming a concentration gradient in 96 well plates. Concentrations were chosen based on the upper limit of each antibiotic defined by the CLSI. The bacterial suspension prepared as per CLSI guidelines was incubated at 37°C for 24 h. The MICs were recorded after 24-h incubation by visual examination. For both methods, the isolates were classified as resistant, intermediate or susceptible based on CLSI breakpoints. The antibiotics chosen in this study are commonly used for treating *P. aeruginosa* infections.

### Genomic Characterization of SPLT Isolates *Pseudomonas aeruginosa*

Multi-locus sequence typing (MLST) and construction of a core genome-based phylogenetic tree using bio-informatic tools was employed to define the genomic relatedness between the 36 serial *P. aeruginosa* isolates and a control panel of 672 *P. aeruginosa* strains.

Genomic DNA from all 36 isolates was extracted using the UltraClean^®^ microbial DNA isolation kit (Qiagen, Netherlands) as per the manufacturer’s instructions. The qualitative and quantitative estimation of the obtained DNA was carried out using the LabChip^®^ GX Touch^TM^ nucleic acid analyzer (PerkinElmer, United States) and Qubit dsDNA BR assay kits (Thermo Fisher Scientific, Waltham, MA, United States). The high-quality DNA was used for whole genome library preparation using S4 Reagent Kit v1.5 by following manufacturer’s instructions. The resultant libraries were sequenced on the NovaSeq 6000 platform (Illumina Inc.) using 2 × 150 bp chemistry at CeGaT (Tübingen, Germany).

### Bio-Informatic Data Processing and Analysis

Raw reads were quality filtered and assembled using the A5-MiSeq pipeline. The assembled contigs were used for further downstream analysis viz. annotation and phylogenetic analysis. The potential for AMR was inferred by annotating the genomes using the PATRIC_3_._6_._2_ server. The virulome and resistome were analyzed using the Virulence Factors Database (VFDB) and Comprehensive Antibiotic Resistance Database (CARD) database on March 12, 2020 and September 9, 2019, respectively (Chen et al., 2005; Alcock et al., 2020). The genomic antibiogram was validated using the BioMérieux EPISEQ^®^ CS tool (v1.0.2)^1^. The genomic antibiogram depicted gene abundance. Gene abundance is the number of times the genes involved in the particular function (e.g., efflux pump conferring resistance) appears in the whole genome sequence. As these genes encoding resistance are usually part of a group, presence or absence of one gene from the group could impact the level of resistance. A core genome-based phylogenetic tree using the FAST tree method was constructed to quantify the genomic relatedness level between the SPLT isolates. This tree was constructed using the parsnp tool (v1.2; Treangen et al., 2014) and was curated using iTOL (v5; Letunic and Bork, 2016). Alternatively, the 7-loci MLST of the 36 *P. aeruginosa* isolates was performed using PubMLST^2^ and BioMérieux EPISEQ^®^ CS to decipher STs and to define relatedness among the isolates (Jolley et al., 2018).

In case of disparity in results between PubMLST and BioMérieux EPISEQ^®^ CS, sequence reads were aligned with the housekeeping gene reference alleles using the BWA MEM algorithm from the Burrows-Wheeler Alignment Tool (BWA; v0.7.17). We used SAMtools (v1.7) to obtain BAM files of the alignments, which were later visualized using the integrative genomics viewer (IGV; ^∗^v2.4.1; Li et al., 2009; Li and Durbin, 2010; Robinson et al., 2011).

### Comparative Genome Analysis of SPLT *Pseudomonas aeruginosa* Isolates

A core genome single nucleotide polymorphism (SNP)-based approach using the parsnp tool was used to compare the sequence of SPLT *P. aeruginosa* isolates to diverse strains of *P. aeruginosa* (*n* = 672) to define the genomic variation between *P. aeruginosa* strains from different niches and geographies. The 672 *P. aeruginosa* strains were obtained from three collections: the private BioMérieux collection (*n* = 219; van Belkum et al., 2015); the Kos collection (*n* = 390; Kos et al., 2015); and the Pirnay collection (*n* = 63; Pirnay et al., 2009). Further, the phylogeny was reconstructed based on the core genomes.

## Results

### Identification and MLST Profiling of the SPLT *Pseudomonas aeruginosa* Isolates

On COS agar plates, the isolates were mucoid and slow growing. Despite the aberrant growth patterns for the strains, the verification of the identification of bacterial isolates using VITEK^®^ MS (BioMérieux) confirmed that all isolates were *P. aeruginosa.* The WGS of the CF isolates generated an average of 50,043,100 ± 7,423,658 (average ± SD) pair-end reads per genome (Supplementary Table 1). *De novo* assembly revealed a mean genome size of 6.36 ± 0.17 mb (mean ± SD) with average coverage of 591× (±91×). Both MLST profiling methods traced four STs covering 18 genomes. However, the remaining 18 genomes (the majority belonging to patients 1, 2, 5, and 6) could not be assigned to any known ST and, hence, represent novel STs. LTx had no impact on the changes in the ST of the isolates. For patient 3, all of the isolates belonged to the same known ST (146), while for patients 1 and 5 they all belonged to novel STs. For patients 2 and 6, some of the isolates pre- and post-transplantation belonged to the same known ST (ST1029 and ST1567, respectively) while others belonged to novel STs. Finally for patient 4, some of the isolates at the time of transplant belonged to the same known sequence type (ST882) as one of the earlier isolates, while others belong to a novel ST identical to the post-LTx isolate (Supplementary Table 1).

Through the BioMérieux EPISEQ^®^ CS analysis, ST235 was assigned to isolates 810, 1,118, and 1,119 which were isolated from patient 6 after LTx. MLST performed by aligning the reads to the reference sequence analysis showed mutation in the *acs*A allele in isolate 1,118. However, no mutation was found in the isolates 810 and 1,119. For our specific set of *P. aeruginosa* isolates, BioMérieux EPISEQ^®^ CS proved to be a more robust MLST tool as it could assign STs to a larger number of isolates.

### Comparison of *Pseudomonas aeruginosa* From Different Collections of Isolates

**FIGURE 1:**
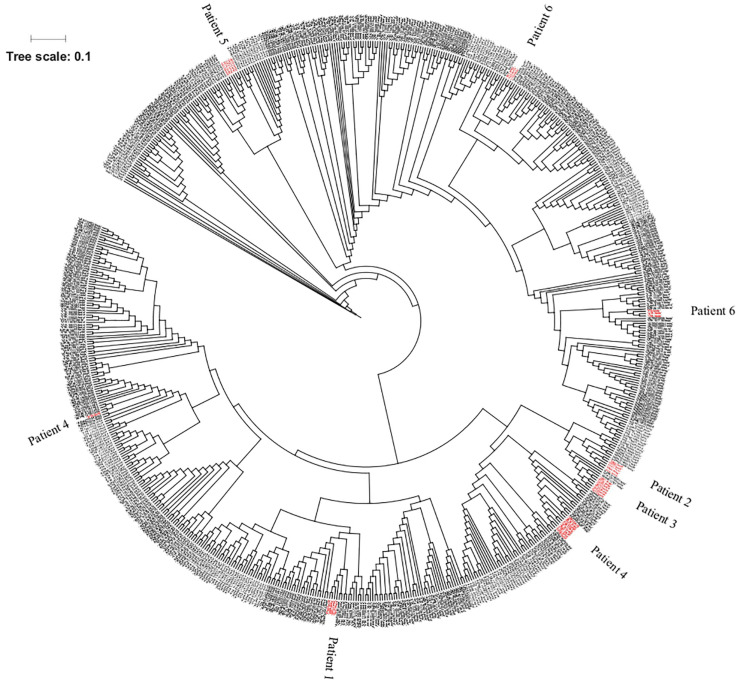
Genotypic similarities between 708 *Pseudomonas aeruginosa* strains at core genome SNP level and genotypic similarities/differences in a SNP based tree. Out of the 708 strains, 36 isolates were from lung transplant patients (in red) and the others belong to BioMérieux collection, Kos collection, and Pirnay collection.

The genomic comparison of the isolates under investigation with the genomes of 672 isolates depicted unique clustering of the SPLT isolates in seven different clades spanning across the range of isolates isolated from diverse niches (Figure 1). However, the SPLT isolates were found to be conserved at the individual patient level, forming a monophyletic clade per patient, except for the case of patient 6 where isolates formed two distinct clades. Clade I contained the isolates 160, 179, and 811 with ST1567 whilst the other clade (clade II) was comprised of isolates 810, 1,118, and 1,119, with ST235 according to BioMérieux EPISEQ CS tool.

### Genomic Analysis and Antibiotic Resistance

**FIGURE 2:**
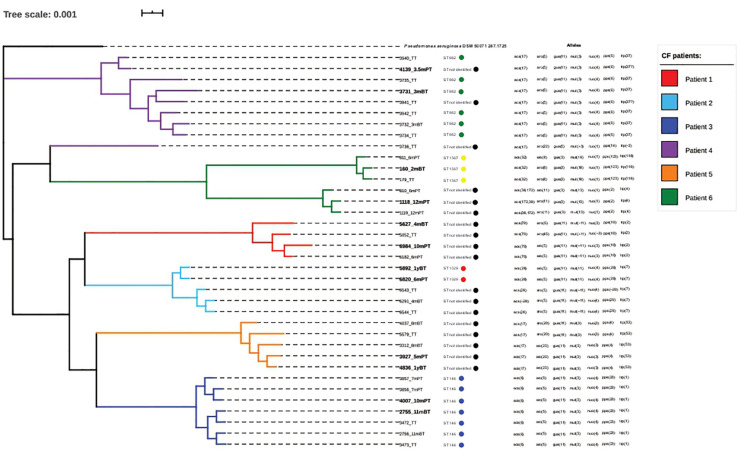
The phylogenetic tree constructed using the whole genome sequence (WGS) of 36 clinical *Pseudomonas aeruginosa* isolates demonstrates the variability between the isolates. Each clade represents one patient. The isolates selected for phenotypic analysis using broth micro-dilution (BMD) assay (in bold) are from two different time points: before and after transplant. The figure also depicts the sequence types and allelic differences among the 36 *P. aeruginosa* isolates from lung transplantation (LTx) patients.

The genomic investigation of the 36 *P. aeruginosa* isolates using WGS revealed differences in the genome architecture sharing ≥98.7% average nucleotide identity (ANI; Supplementary Table 1). Further, a core genome phylogeny developed using the FAST tree method suggested formation of six different clades representing isolates from six different patients. We found intergroup variability with respect to the origin of the isolates. However, clustering did not correlate with time of isolation of the isolates around LTx (Figure 2).

**FIGURE 3:**
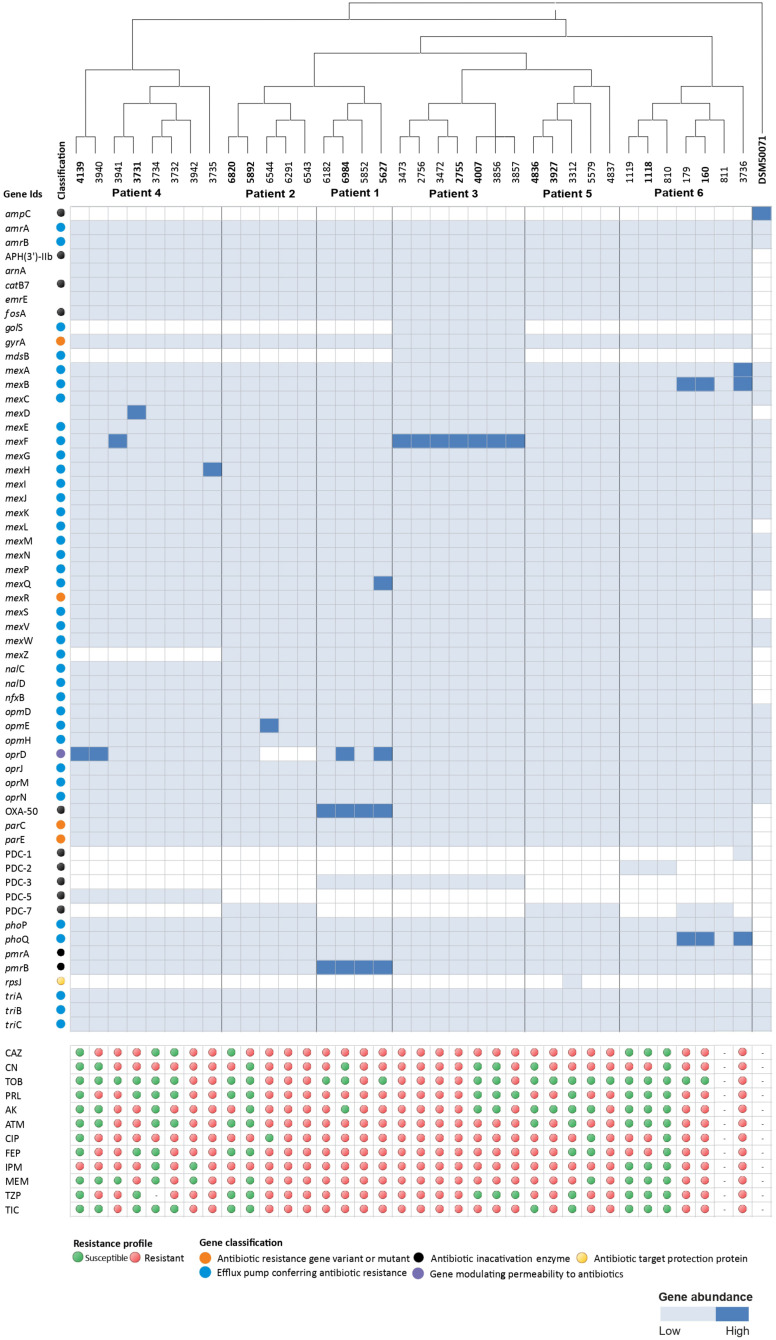
Heatmap-dendrogram analysis of 36 *Pseudomonas aeruginosa* isolates and reference stains depicting the taxonomic relatedness of the isolates isolated across three time points: before LTx, at the time of LTx and after LTx. Furthermore, the qualitative estimates of antibiotic resistance genes (ARGs) were compared against the observed antimicrobial resistance (AMR) phenotypes. AST results are shown by color code red (resistant) and green (susceptible).

The nucleotide sequences of the small subunit (SSU) 16S rRNA gene of all the isolates were extracted and taxonomic identity was reconfirmed using a BLAST search. Further, we noted the presence of 6,150 ± 193 (mean ± SD) coding sequences (CDS) per genome. A subset of the CDSs accounting for conferring antibiotic resistance was derived using CARD, which was in the range of 55 ± 1 resistance CDS per isolate. The genes retrieved from the CARD analysis were mostly classified as beta-lactam resistance genes (*n* = 6), efflux pumps (*n* = 37), an aminoglycoside resistance gene (*n* = 1), other antibiotic inactivation enzymes (*n* = 8), antibiotic resistance gene variants or mutants (*n* = 4), a gene modulating bacterial permeability to antibiotics (*n* = 1), genes altering cell wall charge conferring antibiotic resistance (*n* = 2) and an antibiotic target protection protein gene (*n* = 1; Figure 3).

**FIGURE 4:**
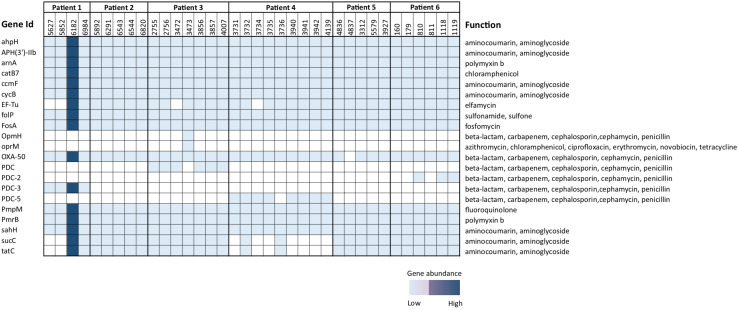
Presence and absence of antimicrobial resistance genes in 36 *Pseudomonas aeruginosa* isolates. Varied degree of presence of genes conferring resistance against drug families was noted. The analysis was done using EPISEQ.

BioMérieux EPISEQ^®^ CS analysis showed the presence of various antibiotic resistance genes (ARGs) in *P. aeruginosa* isolates. We noted different frequencies of genes conferring resistance against drugs or drug families such as aminocoumarin, aminoglycoside (100%), polymyxin B (100%), chloramphenicol (100%), elfamycin (88.89%), sulfonamide, sulfone (100%), fosfomycin (100%), azithromycin, ciprofloxacin, erythromycin, novobiocin, tetracycline (*oprM* gene, 2.78%), beta-lactam, carbapenem, cephalosporin, cephamycin, penicillin (from 97.22% for OXA-50 to 8.33% for PDC-2 or PDC-3), and fluoroquinolone (100%). For strain 3,473 (belonging to patient 3), we noted the prevalence of genes responsible for resistance against the antibiotics azithromycin, chloramphenicol, ciprofloxacin, erythromycin, novobiocin and tetracycline. Interestingly, in contrast to all other isolates, isolate 4,837 (deriving from patient 5) lacked the presence of genes providing resistance against the drug family “beta-lactam, carbapenem, cephalosporin, cephamycin, and penicillin” (Figure 4).

### Virulence Genes

**FIGURE 5:**
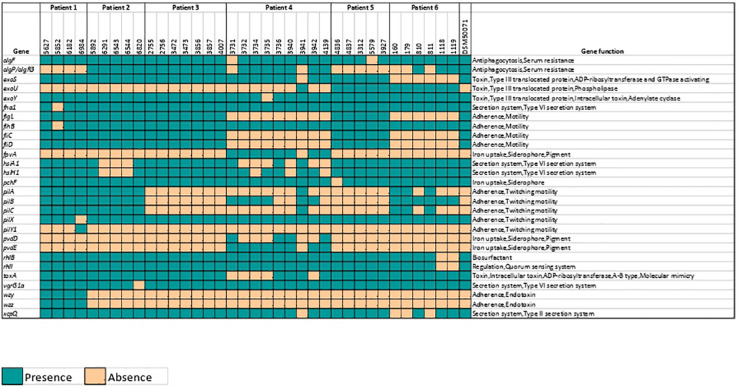
Presence of various virulence factors among 36 *Pseudomonas aeruginosa* isolates. Yellow square indicates absence while green square indicates presence of virulence genes identified using Virulence Factors Database (VFDB).

The investigation of virulence factors in *P. aeruginosa* isolates using VFDB revealed isolates containing various virulence genes ranging from 236 to 266 in number. Genes with no known functionality in the VFDB database were eliminated. The remaining 28 genes were found to be differentially abundant across the genomes off all isolates in the study. The major functional attributes of these 28 genes were adherence (17.8%, relative abundance) and Type VI secretion (14.2%, relative abundance; Figure 5).

### Antibiotic Susceptibility Testing

More than 75% of the isolates were found to be resistant to at least half of the antibiotics under study (Table 2). Isolates 810 (patient 6) and 4,139 (patient 4) were found to be the most susceptible. Tobramycin was found to be effective against 64% of the isolates. Additionally, isolates 3,735 and 3,736 (patient 4), 6,291 and 6,543 (patient 2), 5,852 (patient 1), and 3,473, 3,472, 2,755 and 2,756 (patient 3) were found to be resistant to all the antibiotics tested. Interestingly, all the isolates of an ST belonging to one patient did not always show the same susceptibility pattern (Figure 3).

**TABLE 2:** The MICs (mg/L) of the eight antibiotics against the panel of 12 *P. aeruginosa* isolates by broth micro-dilution (BMD).

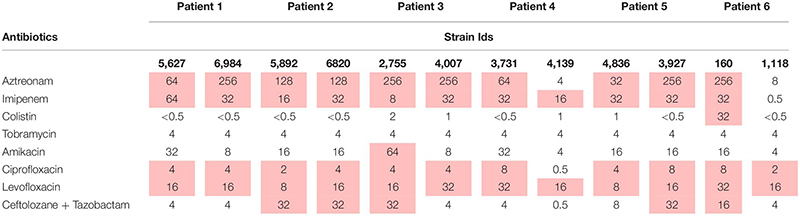

*The strains were categorized as susceptible (white cells) and resistant (red cells) according to the CLSI guidelines on MIC breakpoints.*

We tried to correlate the phenotypic antibiograms to the AMR gene patterns obtained using the CARD analysis, but there were inconsistencies in the results. In patient 3, isolates 4,007 and 3,856 were susceptible to tobramycin, piperacillin and amikacin in contrast to other genotypically similar isolates from the same patient (Figure 3). We noted the unique presence of a β-lactam resistance gene *bla*_*PDC–*__2_ for isolates 810, 1,118, and 1,119, isolated post-transplant from patient 6. Similarly strain 3,312 (8 months before LTx) was the only strain containing a gene for the antibiotic target protection protein *rps*J. Interestingly, 5 months after the LTx this gene seemed to be lost from isolate 3,927. Unlike others, isolates 6,291, 6,543, and 6,544 (4 months before LTx and during LTx, respectively) isolated from patient 2 lacked the gene modulating permeability to antibiotics. The isolates from different time points from patient 4 lacked the presence of *mex*Z gene, which codes for a regulator of an important efflux pump responsible for imparting resistance to aminoglycosides (Figure 3).

## Discussion

Our analysis shows that *P. aeruginosa* isolates from different time points, but from the same patient, contained identical STs suggesting lack of cross infection in the patients under study. These isolates showed resistance to antibiotics in comparison to the susceptible reference isolates. Correct treatment depends on continuous and serial assessment of resistance features. Moreover, in the majority of recurrent infections patients tend to harbor genotypically similar *P. aeruginosa* isolates (Workentine et al., 2013). *P. aeruginosa* is a tough bug to kill and it persists even after prolonged antibiotic treatment as well as lung transplantation (Walter et al., 1997; Mainz et al., 2012; Beaume et al., 2017; Parkins et al., 2018). Therefore, a more frequent follow-up of infection after LTx is necessary to see how the same strain evolves over the course of time.

We determined the taxonomic identity and genomic relatedness of the *P. aeruginosa* isolates. We observed significant genomic homology for the isolates from the same CF patients. Furthermore the MLST typing of the 36 SPLT isolates demonstrated characteristic patient specific unique STs. This observation is supported by the difference seen in the Canadian MLST typing of *P. aeruginosa* (Jeukens et al., 2019). They noted a significant heterogeneity and a need of personalized patient care in the context of CF Core genome phylogeny of the 36 SPLT isolates depicted six clades, one per patient, establishing the fact that patient specific genotypic diversity exists for the *P. aeruginosa* populations during chronic CF infection (Fothergill et al., 2007; Mena et al., 2008; Bragonzi et al., 2009; Wilder et al., 2009; Huse et al., 2010; Mowat et al., 2011; Chung et al., 2012; Workentine et al., 2013; Jiricny et al., 2014). The genotypic and phenotypic divergence among the isolates from different patients could be a result of antibiotic treatment driven evolution of the *P. aeruginosa* populations. Alternative reasons for such evolution could be selective stress due to nutrient and oxygen deficiency, impaired immune response and barrier function of the mucosal linings (Feliziani et al., 2014; Lozano et al., 2018). In CF patients, *P. aeruginosa* causes infections through two ways, i.e., using both adaptive and mutational mechanisms (Lister et al., 2009). The study highlights how isolates from the same patients evolve over time and how it is difficult to correlate the increased MIC with the genotype as there tends to be no clear differences at the genomic level. The resistant nature of the strains could also be related to mutations. Thus, identification of the mutations along with the resistance genes could aid our understanding of increased antibiotic resistance (Khaledi et al., 2019).

Likewise, the assessment of genomic similarity between the SPLT isolates from the other clinical and environmental *P. aeruginosa* strains does not hinder the branching pattern of the SPLT isolates under study except for strains from patients 4 and 6. It is supported by a previous report focusing on indels, recombination or SNPs for definition of inter-patient variability and intra-patient similarity (Huse et al., 2010). Interestingly in patient 6, two clades were formed. One clade consisted of strains before LTx while the other consisted of strains after LTx, which shows how the genotype can alter overtime. Additionally, we saw the spread of the SPLT isolates throughout the phylogenetic tree based on core genes, still showing patient specific clustering. This observation was found to be coherent with the noted difficulties in discriminating CF isolates based on their geography and origin (Jeukens et al., 2019; Pincus et al., 2020).The latter demands further understanding of the emergence of resistance and virulence among these *P. aeruginosa* to avoid or manage lung infections (Cho et al., 2014; Jaillard et al., 2017).

Our investigation of genomic features revealed various AMR and virulence genes. The main characteristics of the virulence genes were related to adhesion and secretion thereby suggesting that these isolates could be biofilm producers. Similarly, the isolates displayed a varied response to the array of antibiotics even though the isolates have homologous MLST types found during the analysis. This might mean that the resistance profile might also be related to host-related factors, which are largely unknown. There have been extensive studies on increased antibiotic resistance in *P. aeruginosa*. Also, the microbes have an ability to integrate exogenous DNA to withstand the selective pressure of antibiotics. Thus, in order to understand these dynamics, there have been various efforts to understand the correlation of genotype and phenotype (e.g., response to antibiotics) by several researchers (Mena et al., 2008; Hazan et al., 2014; Macia et al., 2014; Sousa and Pereira, 2014; Kos et al., 2015; Jaillard et al., 2017; Lozano et al., 2018; Garcia-Nuñez et al.,2020). Furthermore, the MLST profiles derived from two different tools-PubMLST and EPISEQ and the phenotypic antibiotic susceptibility profiles shows disparity. This finding highlights the limitations of the tools and databases and recognizes the impact this could have on predicting resistance and treating infections (Ng et al., 2021). As this study has focused on a very specific set of isolates, from respiratory tract specimens of a few patients from the same hospital, those observed advantages for each tool may be different with a different set of strains.

*Pseudomonas aeruginosa* is a key organism in cases of bronchiectasis leading to increased mortality, as documented in Europe (Ringshausen et al., 2013). The observation that the isolates are genetically alike but show AMR heterogeneity is evident through the recent increase in cases of bronchiectasis by *P. aeruginosa*. Moreover, studies focusing on the United Kingdom population suggested approximately 29% mortality in the bronchiectasis patients (Loebinger et al., 2009). Thus, the British Thoracic Society emphasizes microbiological assessment and personalized treatment to mitigate drug resistance during bronchiectasis due to the multi-drug resistance profile of *P. aeruginosa* (Pasteur et al., 2010).

## Conclusion

Our study sheds light on the lack of intra-patient diversity and phenotypic plasticity of *P. aeruginosa* isolates associated with CF patients undergoing LTx. Most patients with CF carry similar *P. aeruginosa* isolates before, during and after LTx. Genomic similarity between these isolates is observed but they show phenotypic variation. Thus, despite continuous *P. aeruginosa* colonization and infection by a single genotype, these isolates still show varied responses to antibiotics. Our study equally endorses careful assessment for antibiotic susceptibility and high-throughput genomic-level monitoring of antibiotic therapy against *P. aeruginosa* in patients with CF.

## Data Availability Statement

Whole genome sequences for all samples used in this study have been deposited in National Centre for Biotechnology Information (NCBI) and are available under BioProject ID: PRJNA630383 here: https://www.ncbi.nlm.nih.gov/bioproject/PRJNA630383. The isolates for which sequence type were not assigned have been submitted to PubMLST database under the ID: BIGSdb_20200507140502_147974_63511.

## Ethics Statement

All isolates examined in this study were recovered as a result of routine diagnostic procedures. No additional human samples were collected for the purposes of this study. The researchers were supplied with samples of *Pseudomonas* from an archived frozen collection. Researchers were not given access to any data that would allow identification of a particular patient. As the project did not require the use of any human material or any personal data, approval of the local ethics committee was not required.

## Author Contributions

AB, SO, and JP conceived the study. JP and AP collected *P. aeruginosa* isolates from the patients serially and gathered the metadata. RD conducted the microbiological experimentation for *P. aeruginosa* isolates, interpreted the sequence data and wrote the first version of the manuscript. AC, RD, and HG carried out the whole genome sequencing studies. JD assisted in the WGS analysis. All authors discussed the results and edited the manuscript.

## Conflict of Interest

During this study AB, SO, VC, RD, AP, CM, and JD were employes of BioMérieux, a company designing, developing, and marketing tests in the domain of infectious diseases. The company was not involved in the design of the current study and the opinions expressed are those of the authors and may be different from formal company opinions and policies. The remaining authors declare that the research was conducted in the absence of any commercial or financial relationships that could be construed as a potential conflict of interest.

## Footnotes

1 www.biomerieux-episeq.com

2 https://pubmlst.org

## Supplementary Material

Supplementary Figure 1 Genomic similarity among the strain of *P. aeruginosa* isolated from LTx patients, represented by the average nucleotide identity (ANI) as present inter-genomic distances.**Supplementary Table 1 |** Genomic characteristics of *P. aeruginosa* isolates isolated from LTx patients. It includes the attributes of sequencing, MLST types and genomic features viz. ARGs and virulence genes.
