# Supplementary figures and images for: Phenotypic and Genomic Variability of Serial Peri-Lung Transplantation Pseudomonas aeruginosa Isolates From Cystic Fibrosis Patients
Source: Front Microbiol. 2021 Apr 7;12:604555. doi: 10.3389/fmicb.2021.604555 (PMC8058383; doi:10.3389/fmicb.2021.604555)

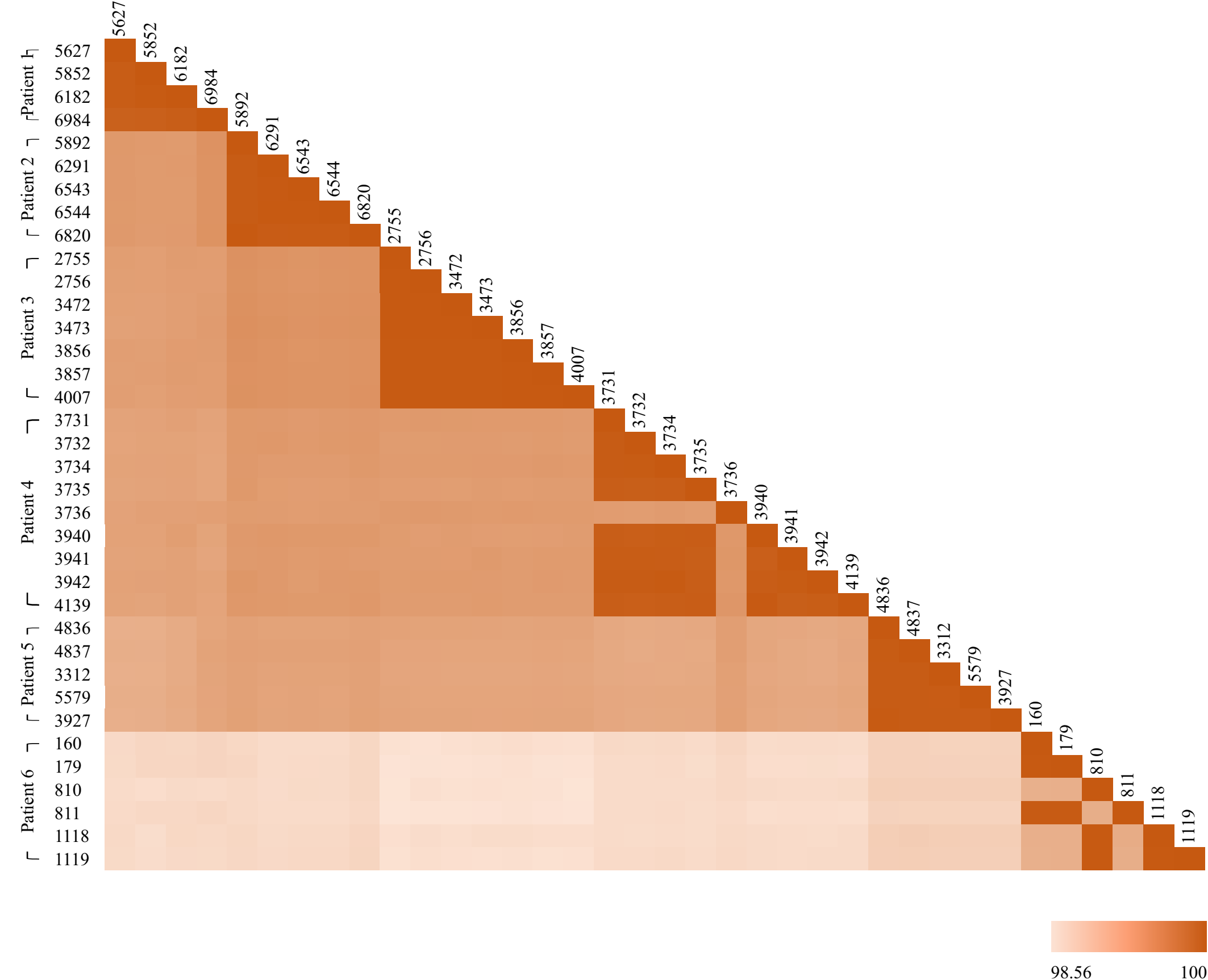

Supplement: Supplementary Figure 1 — Genomic similarity among the strain of P. aeruginosa isolated from LTx patients, represented by the average nucleotide identity (ANI) as present inter-genomic distances.Supplementary Table 1 | Genomic characteristics of P. aeruginosa isolates isolated from LTx patients. It includes the attributes of sequencing, MLST types and genomic features viz. ARGs and virulence genes. [file Data_Sheet_1.PDF]
